# Post-Radiation PET Shows Higher Diagnostic Accuracy in HPV-Negative Head and Neck Cancers

**DOI:** 10.3390/cancers18081237

**Published:** 2026-04-14

**Authors:** Kornél Dános, Angéla Horváth, Emese Kristóf, Imre Uri, Benedek Besenczi, Peter Prekopp, László Tamás, Gábor Polony, Tamás Györke

**Affiliations:** 1 Department of Oto-Rhino-Laryngology, Head and Neck Surgery, Semmelweis University, 1083 Budapest, Hungary; danos.kornel@semmelweis.hu (K.D.);; 2 Medical Imaging Centre, Department of Nuclear Medicine, Semmelweis University, Korányi Sándor Str. 2, 1083 Budapest, Hungary

**Keywords:** head and neck cancers, FDG-PET, neck dissection, residual neck metastasis, follow up

## Abstract

**Background:** Post-treatment evaluation of residual neck disease in head and neck squamous cell carcinoma (HNSCC) is challenging because of treatment-related changes and biological differences between HPV-positive and HPV-negative tumors. **Methods:** We performed a prospective single-center study of 58 node-positive HNSCC patients treated with definitive chemoradiotherapy (CRT) followed by 18F-FDG PET/CT. PET-positive patients underwent neck dissection, while PET-negative patients were followed clinically for at least two years. Diagnostic performance was analyzed by p16 status. **Results:** PET/CT showed high overall accuracy (sensitivity 94%, specificity 83%, PPV 70%, NPV 97%). In p16-negative cases, sensitivity was 93%, specificity 80%, PPV 81%, and NPV 92%. In p16-positive cases, sensitivity and NPV reached 100%, but PPV was only 43%, indicating frequent false positives. Residual disease requiring neck dissection was associated with significantly worse survival (*p* = 0.008). **Conclusions:** PET/CT is reliable for post-treatment assessment, especially in p16-negative HNSCC. In p16-positive cases, careful interpretation and standardized PET/CT criteria are needed to reduce false positives and avoid unnecessary surgery.

## Simple Summary

After chemoradiotherapy (CRT) for head and neck squamous cell carcinoma (HNSCC), accurate detection of residual neck disease is essential to guide further treatment. However, treatment-related changes can mimic persistent tumor on imaging and may lead to unnecessary neck dissection (ND). Biological differences between human papillomavirus (HPV)-positive and HPV-negative tumors may influence imaging performance. In this prospective study of 58 node-positive HNSCC patients, we assessed the diagnostic accuracy of 18F-FDG PET/CT according to p16 status. We confirmed the high overall reliability of PET/CT, particularly in p16-negative disease. In contrast, p16-positive tumors were more frequently associated with false-positive findings. Our results suggest that PET/CT is a valuable tool for post-CRT assessment, and that integrating tumor biology into image interpretation may help reduce overtreatment and improve patient management.

## 1. Introduction

Managing patients after definitive treatment for head and neck squamous cell carcinoma (HNSCC) is often a challenge—especially when it comes to assessing residual nodal disease. Following definitive chemoradiotherapy (CRT), post-treatment imaging plays a critical role in identifying those who may benefit from salvage neck dissection [1].

According to current NCCN (National Comprehensive Cancer Network) guidelines, restaging modalities should include contrast-enhanced CT or MRI for the primary tumor and neck, contrast-enhanced CT/MRI or PET/CT for the neck lymph nodes, and PET/CT or contrast-enhanced chest CT to detect distant metastases. The timing of follow-up imaging depends on clinical suspicion: in the absence of signs of residual disease, imaging is typically recommended 3–4 months post-treatment, while earlier imaging (at 4–8 weeks) is advised if residual tumor is suspected by physical examination.

PET/CT is preferred over conventional imaging during this phase due to higher sensitivity in detecting both residual cervical disease and distant metastases, and it is also beneficial in surgical planning for midline-crossing tumors or contralateral nodal metastases [2]. A previous study with a limited sample size suggests that PET/CT has a strong rule-in value for residual disease, while its negative predictive value remains limited [3]. In contrast, a large-scale study has shown that in patients with N2–N3 disease, PET/CT-guided assessment has demonstrated superiority over routine elective neck dissection [4].

The timing of post-treatment imaging is critical. Early imaging, especially within 12 weeks after completion of therapy, is associated with a high rate of false-positive findings. The optimal imaging window appears to be between 3 and 6 months post-therapy, where a negative PET/CT is strongly associated with improved two-year survival [2].

According to NCCN recommendations, imaging findings should be interpreted in a modality-specific manner. PET/CT plays a central role in decision-making: if it demonstrates clearly resectable, FDG-avid residual nodal disease, neck dissection is recommended. In contrast, CT and MRI are primarily used for anatomical assessment; if these modalities are positive but inconclusive at 8–12 weeks, a follow-up PET/CT performed at least 12 weeks after treatment is recommended. If persistent disease is confirmed on PET/CT, surgical resection is indicated, whereas a negative PET/CT supports close clinical observation [5].

Salvage neck dissection carries considerable morbidity, with higher complication rates than primary surgery [6]. Early postoperative complications may include fistula, wound healing issues, while long-term side effects include chronic shoulder pain and dysfunction, neck fibrosis, and decreased quality of life [7]. In line with findings from a review, the most frequently reported complications include surgical site infection, wound dehiscence, and chyle leak [8]. Patients undergoing both CRT and neck dissection experienced greater pain compared to those who did not undergo surgery [9]. As the extent of surgery is the main predictor of surgical complications [10], appropriate patient selection is essential; therefore, unnecessary operations must be avoided.

These patients have already undergone definitive treatment and often suffer from significant side effects, making the choice between surgical intervention and close follow-up particularly critical. Individuals with positive cervical lymph nodes carry an elevated risk for regional and distant metastases, so the decision at this stage may have a substantial impact on long-term outcomes.

Recent literature on prognostic factors associated with salvage neck dissection suggests that the timing of the neck dissection itself does not bear a significant impact on survival. Rather, the presence of viable metastatic lymph nodes at the time of surgery appears to be the key determinant of outcome. However, in cases of clinically manifest nodal recurrence—such as large, palpable metastases—delaying surgery may increase the risk of inoperability/irresectability, underscoring the importance of timely intervention in selected patients [11].

There are well-established differences between HPV-positive and HPV-negative HNSCC in terms of biology, clinical outcomes, and radiologic features [12]. According to the most recent review and meta-analysis on the topic, significant differences can be found in the diagnostic performance of FDG-PET/CT between HPV-positive and HPV-negative HNSCC. These differences are attributed to the distinct biological behavior and immune response associated with HPV status. FDG-PET/CT sensitivity has been reported to be lower in HPV-positive tumors compared to HPV-negative disease (75% vs. 89%; *p* = 0.01), likely reflecting the higher radiosensitivity of HPV-positive tumors and the longer time required for repopulation of resistant tumor cells to become detectable on early post-treatment PET imaging. Similarly, specificity has been shown to be reduced in HPV-positive tumors (87% vs. 95%; *p* < 0.005), which may be explained by a more pronounced cytotoxic T-cell-mediated immune response, resulting in persistent inflammatory nodal changes. These findings suggest that delayed post-treatment PET/CT imaging in HPV-positive patients may help reduce the detection of treatment-related inflammation and reduce the number of unnecessary neck dissections [13].

Balancing oncologic safety with function preservation is crucial in this patient population. Given these clinical implications, our study aimed to compare the diagnostic performance of PET/CT after CRT between HPV-positive and -negative cancer patients (sensitivity, specificity, positive predictive value (PPV), and negative predictive value (NPV)).

## 2. Materials and Methods

This prospective, single-center study was conducted at the Department of Oto-Rhino-Laryngology, Head and Neck Surgery, Semmelweis University, between 2017 and 2025. The study included 58 patients diagnosed with oropharyngeal, oral cavity, hypopharyngeal, or laryngeal squamous cell carcinoma with regional nodal metastasis (proven with staging primary PET/CT), who received definitive CRT as primary treatment. Inclusion criteria were completed concurrent CRT (using a minimum of one dose of cisplatin 100 mg/m^2^ combined with radiotherapy to a total dose of 66–70 Gy), and post-treatment 18F-FDG PET/CT imaging performed at the Department of Nuclear Medicine of Semmelweis University.

All patients included underwent re-staging PET/CT after CRT. The positive nodal cases underwent salvage comprehensive neck dissection, followed by histological evaluation of the presence/absence of viable metastasis. Pathological workup was done at Semmelweis University, Department of Pathology, Forensic and Insurance Medicine.

Positive PET findings were correlated with histopathological results obtained from salvage neck dissection. In contrast, nodal-negative cases did not undergo surgical verification and were therefore evaluated based on clinical and imaging follow-up for a minimum of two years. Patients without evidence of recurrence during this follow-up period were considered true negative, whereas those who developed regional recurrence were classified as false negative.

Exclusion criteria included: primary tumor located outside the specified head and neck subsites, PET/CT examination performed at an outside institution, non-PET post-treatment evaluation, unavailable p16 staining for oropharyngeal cancer patients or clinical follow-up of less than two years in PET-negative cases.

Patients received 2.5–3.0 MBq/kg of 18F-Fluorodeoxyglucose (18F-FDG) intravenously following a fasting period of at least 6 h. Sixty minutes post-injection, a low-dose, non-contrast-enhanced CT scan and three-dimensional PET emission images were acquired from the jugulum to the mid-thigh. This was followed by an additional dedicated scan of the head and neck region (from the frontal sinus to the aortic arch) with the arms in the down position.

All scans were completed on a hybrid PET/CT scanner (GE Discovery IQ5; GE Healthcare, Milwaukee, WI, USA). Image reconstruction was performed with CT attenuation correction and using a conventional ordered subset expectation maximization (OSEM) algorithm (six iterations and six subsets) and a Bayesian penalized-likelihood reconstruction algorithm with point-spread function modeling (Q.Clear, β = 350). For every scan, a conventional PET image reconstruction without CT attenuation correction was also made. This method helps to rule out misinterpretation of PET-artifacts due to inaccuracies in CT attenuation correction (e.g., CT and PET image misregistration due to patient movement, overestimation of FDG-uptake due to metallic implants on CT).

PET images were analyzed using two software packages available at our institution, both capable of fused PET/CT image visualization and semiquantitative analysis (GE AW Server™ software (version 3.2 Ext. 3.4; GE Healthcare, Milwaukee, WI, USA) and Mediso InterView™ Fusion software (version 3.10.9; Mediso Medical Imaging Systems, Budapest, Hungary)). Visual interpretation of tracer uptake on PET images was made with correlation to anatomical structures on unenhanced CT scan. PET/CT images were interpreted qualitatively, without the use of a formal scoring system or predefined reference values for locoregional tracer uptake (e.g., jugular vein, liver, or mediastinal blood pool activity). Patient oncologic medical history, presence of post-radiotherapy complications and current symptoms were also considered for differential diagnosis. When reporting, true positive findings needed to be distinguished from the tracer accumulation of known possible non-oncologic origin (e.g., normal or abnormally high post-radiotherapy inflammation, ulceration, osteo-/chondronecrosis, sialadenitis, diffuse muscle uptake due to antalgic neck posture, etc.). In general, moderate or intense uptake at the site of primary tumor or nodal metastases, mainly focal and/or asymmetric, counted as a positive finding for residual disease. Indeterminate findings were also reported, in which cases either follow-up scan was needed (in additional 3 months), or specific clinical reasons decided further patient management.

HPV status was assessed using p16 immunohistochemistry (IHC) by detection of the tumor suppressor p16INK4a (Vitro Master Diagnostics, Sevilla, Spain), mouse anti-human p16-INK4A monoclonal antibody [MX007], dilution 1:100). The primary antibody was applied at 37 °C with an incubation time of 10 min. A case was considered p16-positive when at least 70% of tumor cells demonstrated strong combined nuclear and cytoplasmic staining. In accordance with current clinical practice and guidelines, p16 IHC is widely accepted as a reliable surrogate marker for HPV determination in oropharyngeal carcinoma [14]. Alternative methods, such as E6/E7 HPV mRNA detection, HPV DNA detection by polymerase chain reaction (PCR), or in situ hybridization (ISH), were considered; however, p16 IHC was selected due to its feasibility in routine clinical practice [15].

Data was collected from electronic medical records, multidisciplinary tumor board documentation, imaging studies, and histopathology reports. Statistical analysis was performed using descriptive methods and log-rank survival analysis, presented by Kaplan–Meier curves. Significance level was set at *p* < 0.05. All statistical analyses were performed using TIBCO Statistica 14.0.

The study was approved by the Institutional Ethics Committee of Semmelweis University (SE IKEB 105/2014).

## 3. Results

A total of 60 patients were enrolled into our study of which two were excluded because of indeterminate p16 IHC result. Hence a total of 58 patients were included into the analysis, comprising 38 males and 20 females, with a mean age of 60 years (43–83 years). The primary tumor sites were predominantly located in the oropharynx (n = 46), followed by the oral cavity (n = 4), hypopharynx (n = 5), and supraglottic larynx (n = 3). Additional descriptive statistics are presented in Table 1, which follows the classification based on biological behavior, comparing p16-positive oropharyngeal tumors (group 1) to a combined group of p16-negative oropharyngeal, oral cavity, hypopharyngeal, and supraglottic tumors with similar etiopathology (group 2). All tumors were evaluated according to the 7th edition of the TNM classification.

In our cohort, post-treatment 18F-FDG PET/CT demonstrated a sensitivity of 94% and a specificity of 83% in detecting residual neck metastases following chemoradiotherapy. The high negative predictive value (97%) highlights its reliability in ruling out residual disease, whereas the more modest positive predictive value (70%) reflects the ongoing challenge of distinguishing metabolically active post-therapeutic changes from viable tumor tissue (Table 2).

When patients were stratified based on p16 status, a clear difference in diagnostic performance was observed between the two groups. In p16-negative cases (n = 29), PET/CT demonstrated high sensitivity (93%) and negative predictive value (92%), along with acceptable specificity and positive predictive value (80% and 81% respectively), indicating overall balanced accuracy. In contrast, although p16-positive tumors (n = 29) showed excellent sensitivity (100%) and NPV (100%), the positive predictive value dropped markedly to 43%, suggesting a high false-positive rate in this subgroup, despite relatively preserved specificity (85%) (Table 2).

**Figure 1:**
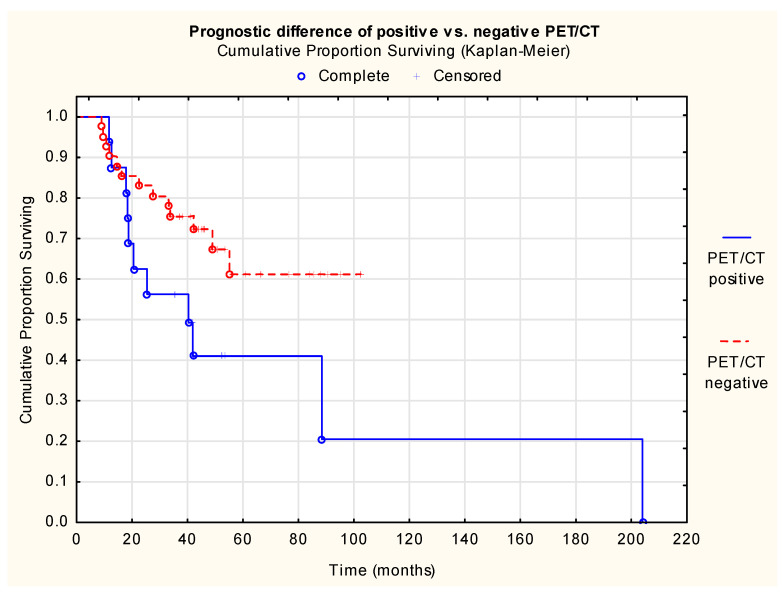
Prognostic difference in positive vs. negative PET/CT. Cumulative proportion surviving (Kaplan–Meier) of patients (Log-Rank Test: WW = 5.963, Sum = 24.110, Var = 5.083, Test statistic = 2.645, *p* = 0.008, n (PET/CT positive) = 17, n (PET/CT negative) = 41, n (patients at risk) = 58).

In the overall study population, log-rank test demonstrated significant survival detriment of patients who had residual disease after CRT requiring salvage neck dissection (*p* = 0.008) (Figure 1).

**Figure 2:**
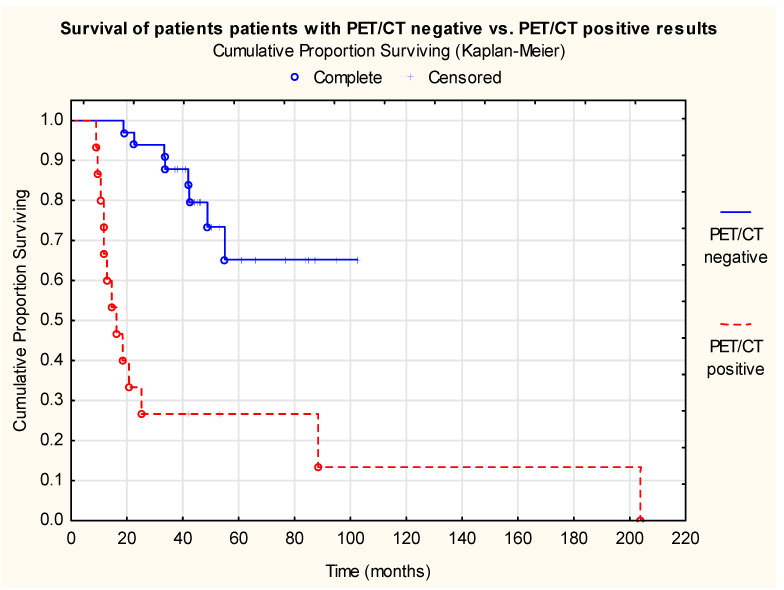
Cumulative proportion surviving (Kaplan–Meier) of patients with PET/CT negative vs. positive results following irradiation (Log-Rank Test: WW = −9.722, Sum = 20.991, Var = 4.712, Test statistic = −4.478, *p* < 0.001, n (true negative) = 34, n (true positive) = 16, n (patients at risk) = 50).

**Figure 3:**
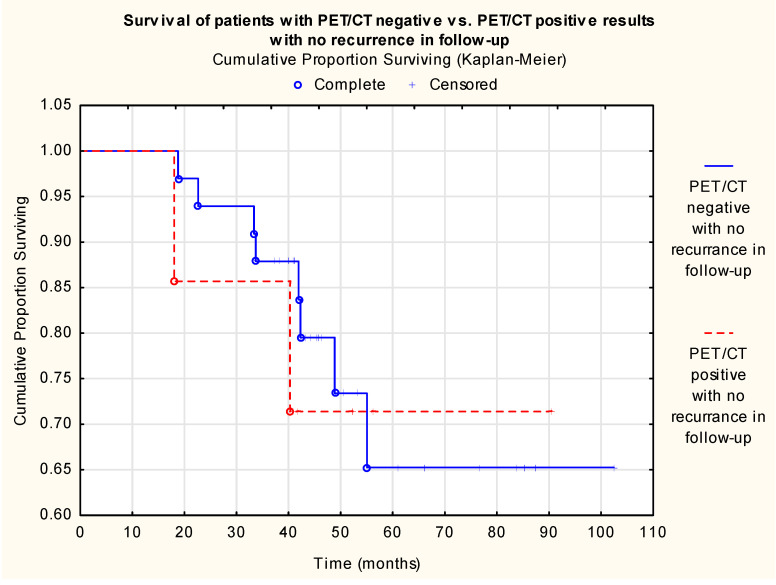
Cumulative proportion surviving (Kaplan–Meier) of patients with PET/CT negative vs. positive results with no recurrence in follow-up (Log-Rank Test: WW = −0.360 Sum = 9.596 Var = 1.4210, Test statistic = −0.302, *p* = 0.763, n (true negative) = 34, n (true positive) = 16, n (patients at risk) = 50).

A significant survival benefit could be proven among true-negative compared to true-positive cases (*p* < 0.001) (Figure 2), while the survival analysis revealed no significant difference between true-negative and false-positive cases (*p* = 0.763), suggesting biological equivalence between the two groups (Figure 3).

Based on our data, the mean interval between the end of CRT and the follow-up PET/CT was 15.7 weeks, with a median of 13.5 weeks (range: 10–33 weeks).

Complications were relatively rare, including wound healing disorder, fistula, and permanent marginal mandibular nerve palsy, in one, one, and two cases, respectively.

## 4. Discussion

Our results are consistent with the systematic review and meta-analysis, which demonstrated that post-treatment FDG-PET/CT is a reliable tool for assessing residual nodal disease after CRT with the sensitivity of 85%, specificity of 93%, confirming high diagnostic accuracy. Similarly, we confirmed that diagnostic performance was lower in HPV-associated cases compared to HPV-negative disease [13].

In our study the reliability of pathologic results as gold standard was validated by survival results: there was no survival difference between true negative and false positive cases whereas true positive cases had significantly worse survival than true negative individuals.

Despite the high negative predictive value of FDG-PET/CT, cytological sampling or biopsy may still be considered to confirm equivocal positive findings. However, it is important to note that neither conventional imaging modalities (CT, MRI, US) nor fine-needle aspiration cytology (FNAC) achieve 100% sensitivity in the evaluation of the neck. Moreover, the rate of false-negative cases has been reported to be relatively high for both approaches: 26.1% with imaging techniques and 33.3% with FNAC [16]. Therefore, neither method alone can reliably confirm or exclude the presence of nodal metastasis, therefore the decisions should be based on individualized clinical assessment.

Prior studies have compared intratherapy and posttherapy FDG-PET/CT and demonstrated that while both have prognostic value, post-treatment imaging appears to provide a more reliable prediction of clinical outcomes [17]. Recent data suggest that PET/CT may influence treatment decision-making even in the postoperative, pre-adjuvant setting [18], thereby contributing to more personalized therapeutic strategies.

In patients with a negative PET/CT scan, observation can be recommended with a high degree of confidence [19], potentially avoiding unnecessary salvage neck dissection with potential complications.

Clear reporting with all the essential information is challenging in post-treatment PET/CT evaluation, particularly for professionals who are not subspecialized in head and neck imaging. More standardized forms of evaluation, like a five-point-scale score system for the likelihood of residual disease (e.g., NI-RADS, Hopkins, modified Deauville criteria, etc.) help remove unnecessary uncertainties from the report, keeping the number of indeterminate cases lower, but highlight the importance of closer follow-up rather than intervention for these patients. However, these criteria are not widely used since there is no consensus on which performs best, and some authors suggest their modifications to create a more specific score system. Although different systems demonstrate similar diagnostic performance, indeterminate findings remain a significant limitation [20]. The use of these criteria, either modified or combined with expert opinion and clinical background, might overcome these drawbacks. Widely understandable reports would be beneficial for less unnecessary surgery and more personalized follow-up imaging [2,21].

The evaluation of the neck following CRT remains a diagnostic challenge, as treatment-induced changes—such as fibrosis, edema, and inflammation [22]—can mimic or mask residual disease on imaging, thereby limiting the specificity of conventional radiologic assessment.

Different factors can affect the accuracy of follow up 18F-FDG PET/CT such as timing, post-treatment changes, inflammation and, according to our observation, p16 positivity in oropharyngeal cancers. Underlying biological differences between HPV-positive and HPV-negative tumors may influence imaging characteristics, as distinct molecular patterns have been described not only within the tumor but also in the surrounding mucosal field [23]. In addition, one possible explanation may be the initially cystic characteristic of HPV-positive lymph node metastases [24].

In HPV-associated oropharyngeal cancers, a longer interval between treatment completion and follow-up imaging may be beneficial; however, this approach does not jeopardize patient safety, as most recurrences occur within the first two years after treatment [25], during which careful imaging follow-up remains essential.

A potential approach proposed by Porceddu et al. would be a second follow up PET/CT around 16 weeks (4–6 weeks after the first re-staging), which could reduce the number of false positive cases [26]. However, in our cohort, the scans were already performed later than the commonly used 12-week interval, and false-positive cases still occurred; therefore, delaying the examination alone would not be a sufficient solution.

In the study published by Zhou et al., HPV-associated HNSCC patients with an equivocal PET/CT result who did not undergo immediate neck dissection had survival outcomes comparable to those who achieved a complete remission [27]. This supports reconsidering clinical decision-making in HPV-associated patients with equivocal PET/CT results.

In order to understand each diagnostic failure, all discordant PET/CT findings (false positives and false negatives) were independently reviewed by an experienced head and neck radiologist with expertise in nuclear medicine. In the single false-negative case, the reviewer noted that the scan was not entirely negative, and additional follow-up imaging had already been recommended at the time of investigation to clarify the finding. Among the seven false-positive cases, five showed no pathological FDG uptake on PET but were considered suspicious on CT due to morphological abnormalities, which led to the decision to proceed with neck dissection. In one case, the PET/CT was performed beyond 11 weeks post-treatment, which may explain the observed diffuse uptake. In another, the reviewer retrospectively confirmed the presence of true uptake suggestive of residual disease. Notably, four of the eight discordant cases occurred in p16-positive oropharyngeal carcinoma, while the other four were in p16-negative or non-oropharyngeal tumors.

Recent studies have demonstrated that the combination of circulating tumor DNA (ctDNA) liquid biopsy with PET improves diagnostic accuracy in HPV-positive HNSCC (sensitivity 100%, specificity 90%) [28]. This could be valuable in equivocal cases, aiding treatment decisions and avoiding overtreatment.

Finally, based on ESMO recommendations [29], patients with unclear imaging results or more complex presentations should ideally be referred to specialized tertiary centers, where interpretation and management benefit from multidisciplinary expertise.

The goal is to maximize oncologic safety while minimizing the impact on quality of life as much as possible. However, according to current national guidelines [30], oncologic control takes priority—even at the cost of performing unnecessary dissections. That is why in our clinical practice, we prioritized oncologic safety in ambiguous cases, which resulted in a higher number of unnecessary neck dissections.

## 5. Conclusions

Overall, PET/CT represents a reliable tool for post-treatment assessment in HNSCC, especially in p16-negative cases, where it can be used confidently to exclude residual disease. In contrast, in p16-positive cases, PET/CT findings require careful interpretation due to the lower positive predictive value and higher false-positive rate. In order to mitigate this diagnostic dilemma, the development of well-defined PET/CT interpretation criteria and evidence-based guidelines would be essential to support clinicians in decision-making and minimize unnecessary salvage surgery.

## Figures and Tables

**Table 1:** Descriptive statistics.

	Total	p16 Positive Oropharynx (Group 1)	All, Except p16 Positive Oropharynx (Group 2)
Tumor origin			
	58 (100%)	29 (50%)	29 (49%)
oral cavity			4 (7%)
p16 neg. oropharynx			17 (29%)
hypopharynx			5 (9%)
supraglottic larynx			3 (5%)
Age at diagnosis			
x < 40	0 (0%)	0 (0%)	0 (0%)
40 ≤ x< 50	8 (14%)	7 (24%)	1 (3%)
50 ≤ x < 60	17 (29%)	8 (28%)	9 (31%)
60 ≤ x < 70	29 (50%)	11 (38%)	18 (62%)
70 ≤ x < 80	3 (5%)	2 (7%)	1 (3%)
80 ≤ x < 90	1 (2%)	1 (3%)	0 (0%)
90 ≤ x < 100	0 (0%)	0 (0%)	0 (0%)
Missing	0 (0%)	0 (0%)	0 (0%)
Mean (in years) ± SD	60.22 ± 8.83	58.59 ± 10.71	61.85 ± 6.22
Sex			
Male	38 (66%)	18 (62%)	20 (69%)
Female	20 (34%)	11 (38%)	9 (31%)
Unknown	0 (0%)	0 (0%)	0 (0%)
Tobacco use			
Current	25 (43%)	8 (28%)	17 (59%)
Former	7 (12%)	3 (10%)	4 (14%)
Never	18 (31%)	12 (41%)	6 (21%)
Unknown	8 (14%)	6 (21%)	2 (7%)
Alcohol consumption			
Current	16 (28%)	5 (17%)	11 (38%)
Former	2 (3%)	1 (3%)	1 (3%)
Never	30 (52%)	17 (59%)	13 (45%)
Unknown	10 (17%)	6 (21%)	4 (14%)
ECOG			
0	43 (74%)	25 (86%)	18 (62%)
1	12 (21%)	4 (14%)	8 (28%)
2	2 (3%)	0 (0%)	2 (7%)
Unknown	1 (2%)	0 (0%)	1 (3%)
Primary tumor stage before irradiation			
1	7 (12%)	5 (17%)	2 (7%)
2	22 (38%)	16 (55%)	6 (21%)
3	11 (19%)	5 (17%)	6 (21%)
4a	12 (21%)	2 (7%)	10 (34%)
4b	6 (10%)	1 (3%)	5 (17%)
Unknown	0 (0%)	0 (0%)	0 (0%)
Node stage before irradiation			
0	not applicable		
1	27 (47%)	19 (66%)	8 (28%)
2a	1 (2%)	0 (0%)	1 (3%)
2b	9 (16%)	3 (10%)	6 (21%)
2c	8 (14%)	3 (10%)	5 (17%)
3	13 (22%)	4 (14%)	9 (31%)
Unknown	0 (0%)	0 (0%)	0 (0%)
TNM stage before irradiation			
1	16 (28%)	16 (55%)	0 (0%)
2	6 (10%)	6 (21%)	0 (0%)
3	10 (17%)	7 (24%)	3 (10%)
4a	14 (24%)	0 (0%)	14 (48%)
4b	12 (21%)	0 (0%)	12 (41%)
Unknown	0 (0%)	0 (0%)	0 (0%)
5 year survival			
yes	11 (19%)	6 (21%)	5 (17%)
no	2 (3%)	0 (0%)	2 (7%)
shorter surveillance	45 (78%)	23 (79%)	22 (76%)
PET/CT nodal tumor stage following irradiation			
positive	23 (40%)	7 (24%)	16 (55%)
negative	35 (60%)	22 (76%)	13 (45%)
Unknown	0 (0%)	0 (0%)	0 (0%)
follow up PET/CT result			
true positive	16 (28%)	3 (10%)	13 (43%)
true negative	34 (59%)	22 (76%)	12 (43%)
false positive	7 (12%)	4 (14%)	3 (10%)
false negative	1 (2%)	0 (0%)	1 (3%)
Unknown	0 (0%)	0 (0%)	0 (0%)

Note: SD: Standard Deviation; ECOG: Eastern Cooperative Oncology Group; TNM: Tumor–Node–Metastasis.

**Table 2:** Percentage values for PET/CT.

	Sensitivity	Specificity	Positive Predictive Value	Negative Predictive Value
total	94	83	70	97
p16 negative	93	80	81	92
p16 positive	100	85	43	100

## Data Availability

The datasets used and analyzed during the current study are available from the corresponding author on reasonable request.

## Abbreviations

The following abbreviations are used in this manuscript:

CRT	Chemoradiotherapy
CT	Computed Tomography
ctDNA	Circulating Tumor DNA
ECOG	Eastern Cooperative Oncology Group
ESMO	European Society for Medical Oncology
FDG	Fluorodeoxyglucose
FNAC	Fine-Needle Aspiration Cytology
Gy	Gray
HNSCC	Head and Neck Squamous Cell Carcinoma
HPV	Human Papillomavirus
IHC	Immunohistochemistry
ISH	In Situ Hybridization
MBq	Megabecquerel
mRNA	Messenger Ribonucleic Acid
MRI	Magnetic Resonance Imaging
NCCN	National Comprehensive Cancer Network
ND	Neck Dissection
NI-RADS	Neck Imaging Reporting and Data System
NPV	Negative Predictive Value
OSEM	Ordered Subset Expectation Maximization
p16	Cyclin-Dependent Kinase Inhibitor 2A Protein
PCR	Polymerase Chain Reaction
PET	Positron Emission Tomography
PET/CT	Positron Emission Tomography/Computed Tomography
PPV	Positive Predictive Value
SD	Standard Deviation
TNM	Tumor–Node–Metastasis
US	Ultrasound

## References

[1] Strohl M.P., Ha P.K., Flavell R.R., Yom S.S. (2021). PET/CT in Surgical Planning for Head and Neck Cancer. Semin. Nucl. Med..

[2] Jain S., Takalkar A.M., Hall L.T., Hall L.T. (2023). Molecular Imaging of Head and Neck Cancers. Molecular Imaging and Therapy [Internet].

[3] Dejaco D., Uprimny C., Widmann G., Riedl D., Moser P., Arnold C., Steinbichler T.B., Kofler B., Schartinger V.H., Virgolini I. (2020). Response evaluation of cervical lymph nodes after chemoradiation in patients with head and neck cancer—Does additional [18F]FDG-PET-CT help?. Cancer Imaging.

[4] Mehanna H., Wong W.-L., McConkey C.C., Rahman J.K., Robinson M., Hartley A.G.J., Nutting C., Powell N., Al-Booz H., Robinson M. (2016). PET-CT surveillance versus neck dissection in advanced head and neck cancer. N. Engl. J. Med..

[5] National Comprehensive Cancer Network (2025). NCCN Clinical Practice Guidelines in Oncology: Head and Neck Cancers (Version 1.2025). https://www.nccn.org/guidelines.

[6] Molteni G., Comini L., Le Pera B., Bassani S., Ghirelli M., Martone A., Mattioli F., Nocini R., Santoro R., Spinelli G. (2021). Salvage neck dissection for isolated neck recurrences in head and neck tumors: Intra and postoperative complications. J. Surg. Oncol..

[7] Navran A., Gouw Z.A.R., Klop W.M., Schreuder W.H., Donswijk M.L., Owers E., de Boer J.P., Smit L., Karssemakers L., Brekel M.v.D. (2025). Postoperative complications following salvage neck dissection after (chemo)radiotherapy for head and neck squamous cell carcinoma: Which patients are at high risk?. BMC Cancer.

[8] Henneman R., Schats W., Karakullukcu M.B., Brekel M.W.v.D., Smeele L.E., Lohuis P.F., van der Hage J.A., Al-Mamgani A., Balm A.J. (2021). Surgical site complications of post-chemoradiotherapy neck dissection: Urgent need for standard registration. Eur. J. Surg. Oncol..

[9] Donatelli-Lassig A.A., Duffy S.A., Fowler K.E., Ronis D.L., Chepeha D.B., Terrell J.E. (2008). The effect of neck dissection on quality of life after chemoradiation. Otolaryngol. Neck Surg..

[10] Bovenkamp K.v.D., Noordhuis M., Oosting S., van der Laan B., Roodenburg J., Bijl H., Halmos G., Plaat B. (2017). Clinical outcome of salvage neck dissections in head and neck cancer in relation to initial treatment, extent of surgery and patient factors. Clin. Otolaryngol..

[11] Ganly I., Bocker J., Carlson D.L., D’Arpa S., Coleman M., Lee N., Pfister D.G., Shah J.P., Patel S.G. (2010). Viable tumor in postchemoradiation neck dissection specimens as an indicator of poor outcome. Head Neck.

[12] Martinelli C., Ercoli A., Parisi S., Iatì G., Pergolizzi S., Alfano L., Pentimalli F., De Laurentiis M., Giordano A., Cortellino S. (2025). Molecular Mechanisms and Clinical Divergences in HPV-Positive Cervical vs. Oropharyngeal Cancers: A Critical Narrative Review. BMC Med..

[13] Helsen N., Wyngaert T.V.D., Carp L., Stroobants S. (2018). FDG-PET/CT for treatment response assessment in head and neck squamous cell carcinoma: A systematic review and meta-analysis of diagnostic performance. Eur. J. Nucl. Med..

[14] Seiwert T.Y., Zuo Z., Keck M.K., Khattri A., Pedamallu C.S., Stricker T., Brown C., Pugh T.J., Stojanov P., Cho J. (2015). Integrative and Comparative Genomic Analysis of HPV-Positive and HPV-Negative Head and Neck Squamous Cell Carcinomas. Clin. Cancer Res..

[15] Taberna M., Mena M., Pavón M.A., Alemany L., Gillison M.L., Mesía R. (2017). Human papillomavirus-related oropharyngeal cancer. Ann. Oncol..

[16] Horváth A., Prekopp P., Polony G., Székely E., Tamás L., Dános K. (2020). Accuracy of the preoperative diagnostic workup in patients with head and neck cancers undergoing neck dissection in terms of nodal metastases. Eur. Arch. Oto-Rhino-Laryngol..

[17] Sheikhbahaei S., Ahn S.J., Moriarty E., Kang H., Fakhry C., Subramaniam R.M. (2015). Intratherapy or Posttherapy FDG PET or FDG PET/CT for Patients with Head and Neck Cancer: A Systematic Review and Meta-analysis of Prognostic Studies. Am. J. Roentgenol..

[18] Courtney P.T., Casillas J.E.J., Liu E.Y., Sim M.-S., Chau L.W., Lopez-Chicas R.E., John M.A.S., Abemayor E., Blackwell K.E., Chhetri D.K. (2025). Impact of postoperative FDG-PET/CT on adjuvant head and neck cancer treatment. JNCI Cancer Spectr..

[19] Navran A., Kayembe M.T., Gouw Z.A., Vogel W.V., Karssemakers L., de Boer J.P., Donswijk M.L., Schreuder W.H., Owers E., Brekel M.v.D. (2024). FGD-PET/CT three months after (chemo)radiotherapy for head and neck squamous cell carcinoma spares considerable number of patients from a salvage neck dissection. Radiother. Oncol..

[20] Zhong J., Sundersingh M., Dyker K., Currie S., Vaidyanathan S., Prestwich R., Scarsbrook A. (2020). Post-treatment FDG PET-CT in head and neck carcinoma: Comparative analysis of 4 qualitative interpretative criteria in a large patient cohort. Sci. Rep..

[21] Patel Z., Schroeder J.A., Bunch P.M., Evans J.K., Steber C.R., Johnson A.G., Farris J.C., Hughes R.T. (2022). Discordance Between Oncology Clinician–Perceived and Radiologist-Intended Meaning of the Postradiotherapy Positron Emission Tomography/Computed Tomography Freeform Report for Head and Neck Cancer. Arch. Otolaryngol. Neck Surg..

[22] Brahmbhatt S., Overfield C.J., Rhyner P.A., Bhatt A.A. (2024). Imaging of the Posttreatment Head and Neck: Expected Findings and Potential Complications. Radiol. Imaging Cancer.

[23] Orosz E., Gombos K., Petrevszky N., Csonka D., Haber I., Kaszas B., Toth A., Molnar K., Kalacs K., Piski Z. (2020). Visualization of mucosal field in HPV positive and negative oropharyngeal squamous cell carcinomas: Combined genomic and radiology based 3D model. Sci. Rep..

[24] Goldenberg D., Begum S., Westra W.H., Khan Z., Sciubba J., Pai S.I., Califano J.A., Tufano R.P., Koch W.M. (2008). Cystic lymph node metastasis in patients with head and neck cancer: An HPV-associated phenomenon. Head Neck.

[25] Iocca O., Campo F., Schilling C., Payne K., Di Maio P. (2025). Current evidence and future directions in the surveillance of HPV-positive oropharyngeal carcinoma. Expert Rev. Anticancer Ther..

[26] Porceddu S.V., Pryor D.I., Burmeister E., Burmeister B.H., Poulsen M.G., Foote M.C., Panizza B., Coman S., McFarlane D., Coman W. (2011). Results of a prospective study of positron emission tomography–directed management of residual nodal abnormalities in node-positive head and neck cancer after definitive radiotherapy with or without systemic therapy. Head Neck.

[27] Zhou S., Rulach R., Hendry F., Stobo D., James A., Dempsey M.-F., Grose D., Lamb C., Schipani S., Rizwanullah M. (2020). Positron Emission Tomography-Computed Tomography Surveillance after (Chemo)Radiotherapy in Advanced Head and Neck Squamous Cell Cancer: Beyond the PET-NECK Protocol. Clin. Oncol..

[28] Bola S., Cutts A., Vavoulis D., Shrivastava M., Bhuva S., Schuh A., Shah K., Winter S.C., Taylor J.C. (2025). Circulating tumour DNA to augment PET-CT in determining clinical outcome after head and neck cancer treatment. Eur. J. Cancer.

[29] Machiels J.-P., Leemans C.R., Golusinski W., Grau C., Licitra L., Gregoire V. (2020). Squamous cell carcinoma of the oral cavity, larynx, oropharynx and hypopharynx: EHNS–ESMO–ESTRO Clinical Practice Guidelines for diagnosis, treatment and follow-up. Ann. Oncol..

[30] Egészségügyi Szakmai Kollégium (2023). Egészségügyi Szakmai Irányelv—Szájgarat Daganatok. https://www.neak.gov.hu/felso_menu/szakmai_oldalak/szakmai_iranyelvek/szakmai_iranyelvek.

